# Supplementary material for: Semaphorin Receptors Antagonize Wnt Signaling Through Beta-Catenin Degradation
Source: bioRxiv. 2024 May 29:2024.05.29.596372. Preprint. [Version 1] doi: 10.1101/2024.05.29.596372 (PMC11160715; doi:10.1101/2024.05.29.596372)
Supplement: Supplement 1 [file NIHPP2024.05.29.596372v1-supplement-1.pdf]

bioRxiv preprint doi: https://doi.org/10.1101/2024.05.29.596372; this version posted May 29, 2024. The copyright holder for this preprint (which was not certified by peer review) is the author/funder, who has granted bioRxiv a license to display the preprint in perpetuity. It is made available under aCC-BY 4.0 International license.

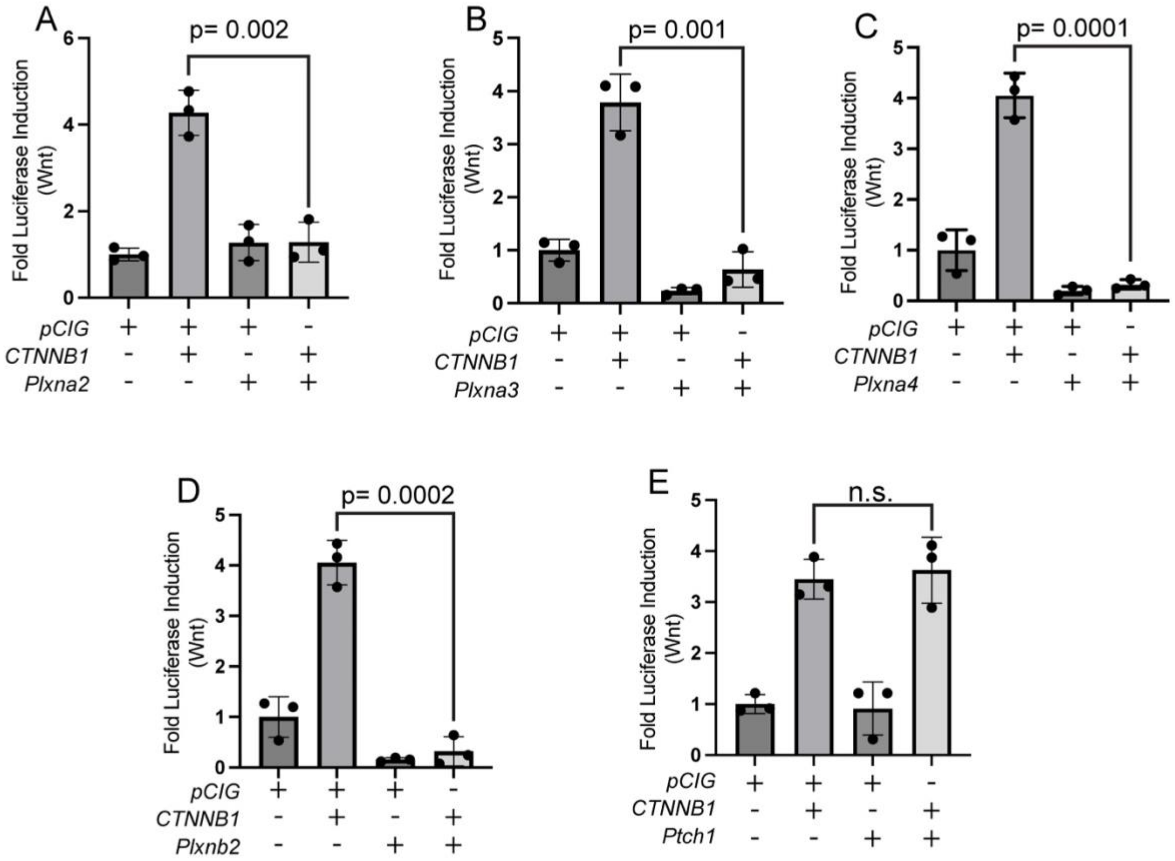

Figure S1. PLXNA and PLXNB subfamily members antagonize Wnt signaling in NIH/3T3 fibroblasts. (**A-E**) Wnt-dependent luciferase reporter activity was measured in NIH/3T3 cells transfected with the indicated plasmids. Data points indicate technical replicates. Data are

bioRxiv preprint doi: https://doi.org/10.1101/2024.05.29.596372; this version posted May 29, 2024. The copyright holder for this preprint (which was not certified by peer review) is the author/funder, who has granted bioRxiv a license to display the preprint in perpetuity. It is made available under aCC-BY 4.0 International license.

representative of at least three biological replicates. Data are reported as mean fold change +/- S.D., with p-values calculated using two-tailed Student's t-test. n.s., not significant.

bioRxiv preprint doi: https://doi.org/10.1101/2024.05.29.596372; this version posted May 29, 2024. The copyright holder for this preprint (which was not certified by peer review) is the author/funder, who has granted bioRxiv a license to display the preprint in perpetuity. It is made available under aCC-BY 4.0 International license.

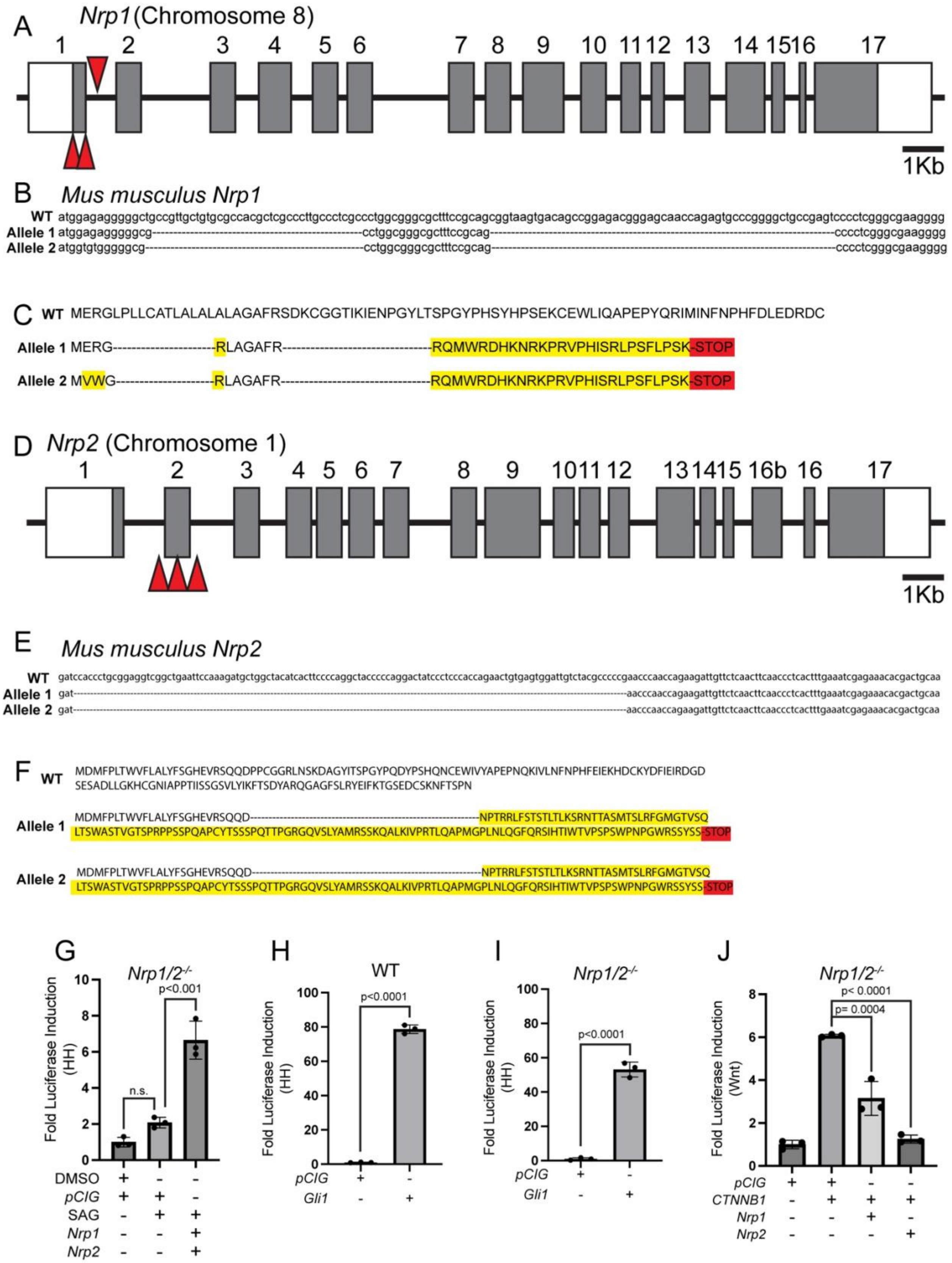

bioRxiv preprint doi: https://doi.org/10.1101/2024.05.29.596372; this version posted May 29, 2024. The copyright holder for this preprint (which was not certified by peer review) is the author/funder, who has granted bioRxiv a license to display the preprint in perpetuity. It is made available under aCC-BY 4.0 International license.

Figure S2. Generation of *Nrp1$^{-/-}$;Nrp2$^{-/-}$* NIH/3T3 cells. (**A,D**) Mouse *Nrp1* (**A**) or *Nrp2* (**D**) locus, including exons (non-coding, white boxes; coding, gray boxes) and introns (lines), with red triangles indicating the target sites of CRISPR gRNAs. Scale bar = 1Kb. (**B, E**) Nucleotide sequence alignment of the WT *Nrp1* (**B**) or *Nrp2* (**E**) sequence and CRISPR-edited alleles. Dashed lines indicate CRISPR-edited nucleotide deletions. (**C, F**) Amino acid sequence alignment of WT and disrupted NRP1 (**C**) or NRP2 (**F**) proteins encoded by the CRISPR-edited *Nrp1* alleles. Dashed lines indicate amino acid deletions; yellow highlighted amino acids represent missense mutations; red highlighting denotes a premature stop codon leading to a truncated protein. (**G**) *Nrp1$^{-/-}$Nrp2$^{-/-}$* NIH/3T3 cells were transfected with the indicated plasmids and HH signaling was measured by luciferase assay. (**H-I**) WT or *Nrp1$^{-/-}$Nrp2$^{-/-}$* NIH/3T3 cells were transfected with the indicated plasmids, and HH signaling was measured by luciferase assay. (**J**) *Nrp1$^{-/-}$Nrp2$^{-/-}$* NIH/3T3 cells were transfected with the indicated plasmids and Wnt signaling levels were measured by luciferase assay. Data are representative of at least three biological replicates. Data are reported as mean fold change +/- S.D., with p-values calculated using two-tailed Student’s t-test. n.s., not significant.

bioRxiv preprint doi: https://doi.org/10.1101/2024.05.29.596372; this version posted May 29, 2024. The copyright holder for this preprint (which was not certified by peer review) is the author/funder, who has granted bioRxiv a license to display the preprint in perpetuity. It is made available under aCC-BY 4.0 International license.

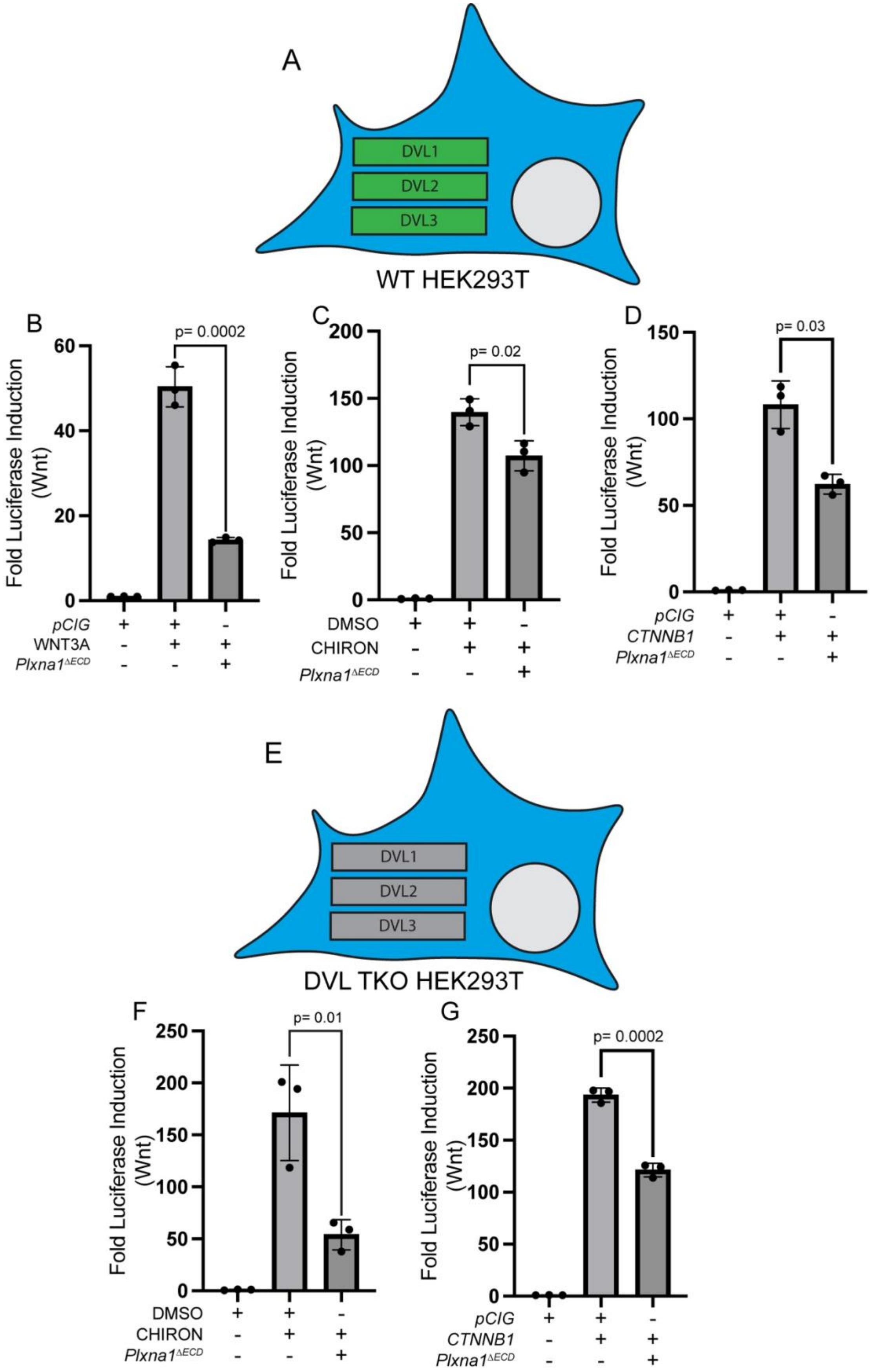

631

bioRxiv preprint doi: https://doi.org/10.1101/2024.05.29.596372; this version posted May 29, 2024. The copyright holder for this preprint (which was not certified by peer review) is the author/funder, who has granted bioRxiv a license to display the preprint in perpetuity. It is made available under aCC-BY 4.0 International license.

Figure S3. PLXN antagonizes Wnt signaling in HEK293T cells in a DVL-independent manner. (**A, E**) Schematic representation of WT or *DVL*$^{-/-}$;*DVL2*$^{-/-}$;*DVL3*$^{-/-}$ (DVL TKO) HEK293T cells. (**B-D, F-G**) Wnt-dependent luciferase reporter activity was measured in WT or DVL TKO HEK293T cells transfected with the indicated plasmids and either left untreated or treated with WNT3A conditioned media, DMSO, or CHIRON. Data are representative of at least three biological replicates. Data are reported as mean fold change +/- S.D., with p-values calculated using two-tailed Student's t-test. n.s., not significant.

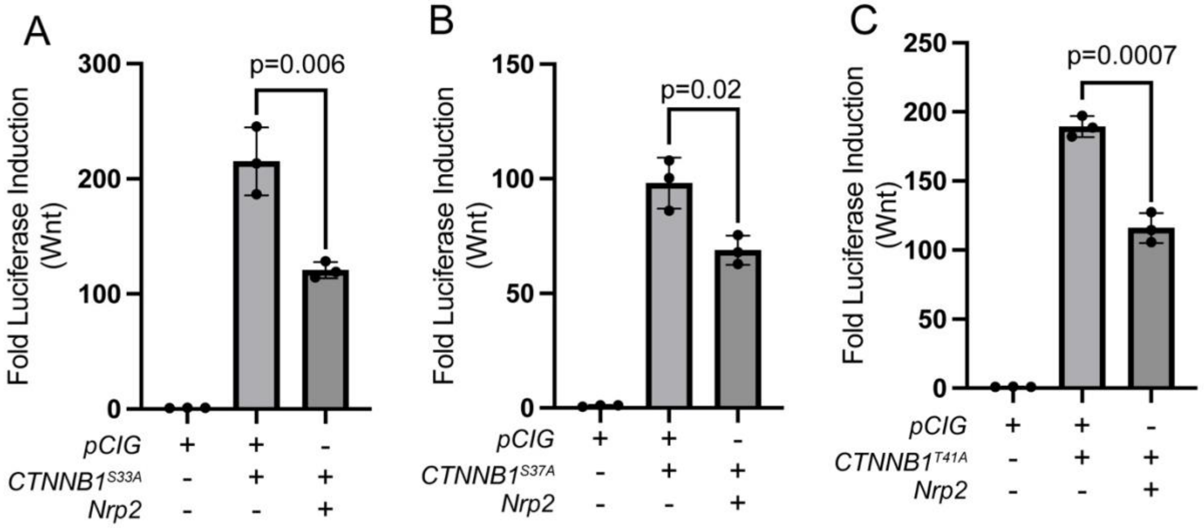

Figure S4. Individual GSK3B phosphorylation site mutants do not affect NRP-mediated Wnt repression. (**A-C**) HEK293T cells were transfected with the indicated plasmids and Wnt signaling levels were measured by luciferase assay. Data are representative of at least three biological replicates. Data are reported as mean fold change +/- S.D., with p-values calculated using two-tailed Student's t-test. n.s., not significant.

bioRxiv preprint doi: https://doi.org/10.1101/2024.05.29.596372; this version posted May 29, 2024. The copyright holder for this preprint (which was not certified by peer review) is the author/funder, who has granted bioRxiv a license to display the preprint in perpetuity. It is made available under aCC-BY 4.0 International license.

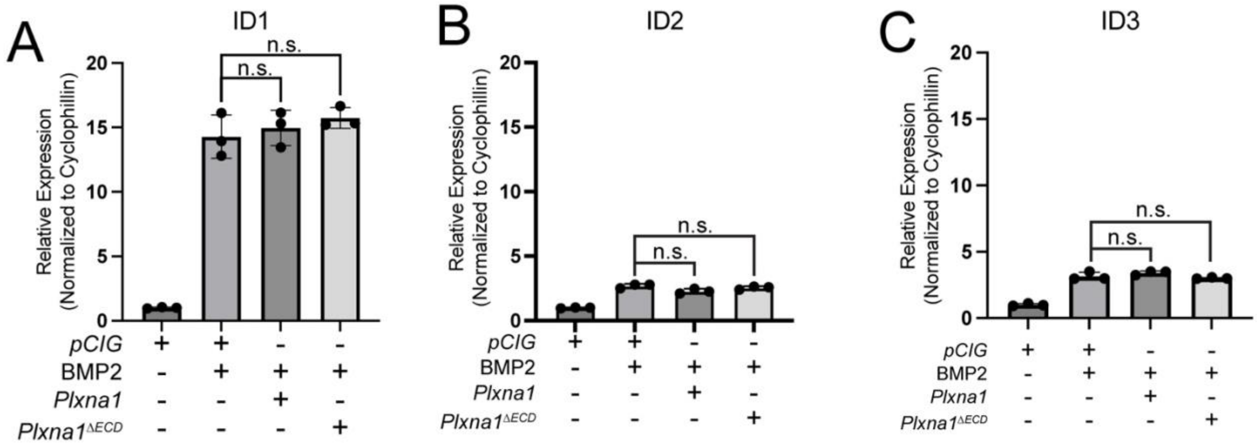

Figure S5. BMP signaling is not modulated by PLXNs. (**A-C**) qRT-PCR analysis of *Id1, Id2,* and *Id3* expression following transfection of NIH/3T3 cells with the indicated plasmids and treatment with BMP2 for 24 hours. Data points indicate technical replicates. Fold changes were determined using the ΔΔCT method normalized to *Cyclophilin*. Data are representative of at least three biological replicates. Data are reported as mean fold change +/- S.D., with p-values calculated using two-tailed Student's t-test. n.s., not significant.
